# Goserelin 3-month depot shows non-inferiority to the monthly formulation in U.S. patients with premenopausal breast cancer: a real-world evidence study

**DOI:** 10.1007/s10549-025-07656-z

**Published:** 2025-03-06

**Authors:** Kelly E. McCann, Noran Osman, Joan Cannon, Lonnie Brent, Yuexi Wang, Jon Tepsick, Prithviraj Vikramsinh Mandora, Vincent Miller, Nancy Martin, Virginia G. Kaklamani

**Affiliations:** 1 Department of Medicine, Division of Hematology and Oncology, David Geffen School of Medicine, University of California los Angeles, Los Angeles, CA USA; 2 TerSera Therapeutics LLC, Deerfield, IL USA; 3 ConcertAI, LLC, Cambridge, MA USA; 4 University of Texas Health Science Center San Antonio, San Antonio, TX USA

**Keywords:** Breast cancer, Event-free survival, Gonadotropin-releasing hormone agonists (GnRHa), Goserelin, Premenopausal, Real-world evidence

## Abstract

**Purpose:**

Clinical trials demonstrated every 3-month goserelin 10.8 mg to be non-inferior to monthly goserelin 3.6 mg in premenopausal patients with ER-positive breast cancer. However, real-world studies comparing 3-month goserelin 10.8 mg with monthly goserelin 3.6 mg are scarce.

**Methods:**

Electronic medical records from the ConcertAI Patient360™ database were analyzed in U.S. patients exposed to goserelin 3.6 mg or 10.8 mg post-breast cancer diagnosis. Inverse probability of treatment weighting (IPTW) was used to ensure the comparability between the two cohorts (goserelin 3.6 mg and goserelin 10.8 mg). The non-inferiority of goserelin 10.8 mg compared with goserelin 3.6 mg was assessed by 12-month real-world event-free survival (rwEFS) rates (− 15% margin) for the overall group of patients and separately for patients with early-stage/locally advanced and metastatic breast cancer.

**Results:**

A total of 575 patients received goserelin 3.6 mg and 123 received goserelin 10.8 mg. Goserelin 10.8 mg was non-inferior to goserelin 3.6 mg based on observed 12-month rwEFS rates (79.2% versus 76.6%, respectively; treatment difference 2.7%). Goserelin 10.8 mg was observed to be non-inferior in patients who initiated goserelin in early-stage/locally advanced (treatment difference − 2.3%) and metastatic (treatment difference 10.4%) breast cancer.

**Conclusion:**

This real-world analysis indicates that 3-month goserelin 10.8 mg is non-inferior to monthly 3.6 mg among premenopausal women with breast cancer in terms of 12-month rwEFS rate. These findings may support the use of the 3-month goserelin 10.8 mg as an alternative treatment option to monthly goserelin 3.6 mg for this patient population.

**Supplementary Information:**

The online version contains supplementary material available at 10.1007/s10549-025-07656-z.

## Introduction

Over three-quarters of breast cancer cases are estrogen receptor-positive (ER +) [1]. In premenopausal patients with ER + breast cancer, tumor growth can be decreased by reducing estrogen production via ovary removal, ovarian ablation, or the administration of a gonadotropin-releasing hormone agonist (GnRHa) [2]. Goserelin, a GnRHa, is recommended in the current NCCN Clinical Practice Guidelines in Oncology (NCCN Guidelines®) for ovarian function suppression with adjuvant endocrine therapy and start of chemotherapy (neoadjuvant or adjuvant) in premenopausal patients with hormone receptor-positive breast cancer who are at high risk of recurrence [3].

Patients with breast cancer who received goserelin had a decreased risk of all-cause mortality and breast cancer related-mortality as well as a lower risk of disease recurrence and disease progression [4, 5]. The efficacy and safety of goserelin, with or without concomitant endocrine therapy, in premenopausal and perimenopausal women with ER + advanced breast cancer are well documented [6–9].

The goserelin 3.6 mg implant is administered every 4 weeks and has been approved by the United States (U.S.) Food and Drug Administration (FDA) for decades for premenopausal and perimenopausal patients with breast cancer [10]. The goserelin 10.8 mg implant is administered once every 12 weeks and may provide a more convenient treatment option for patients due to its less frequent administration schedule. However, goserelin 10.8 mg is currently not approved by the U.S. FDA for patients with breast cancer [11]. The formulation was approved by Health Canada in May 2024 for patients with ER + breast cancer [12].

In addition to a less frequent administration schedule, clinical trials have shown that goserelin 10.8 mg had similar outcomes as goserelin 3.6 mg in premenopausal women with ER + breast cancer. A Phase 2 clinical trial found that goserelin 10.8 mg was non-inferior for serum estrogen suppression compared with goserelin 3.6 mg [13]. A Phase 3 trial conducted in Asia showed that goserelin 10.8 mg was non-inferior compared with goserelin 3.6 mg for progression-free survival at 24 weeks (61.5% versus 60.2%; treatment difference 1.3%) in patients with advanced breast cancer [14]. Although these studies have compared the outcomes of goserelin 3.6 mg and goserelin 10.8 mg in clinical trials, no U.S. studies, to our knowledge, have examined these outcomes of 3-month goserelin 10.8 mg and the monthly goserelin 3.6 mg in the real-world setting.

The aim of this study was to provide insights into the utility of goserelin 10.8 mg in patients with breast cancer. The primary objective of this study was to evaluate if the real-world event-free survival (rwEFS) in patients receiving goserelin 10.8 mg was non-inferior to goserelin 3.6 mg in the real-world setting. The study also sought to examine patient characteristics and real-world clinical outcomes among patients with breast cancer who received goserelin 3.6 mg and 10.8 mg.

## Methods

### Study population

This retrospective, non-interventional study used electronic medical record (EMR) data collected from geographically diverse oncology practice locations within the U.S. and included in the ConcertAI Patient360™ Breast Cancer dataset. The ConcertAI Patient360™ dataset comprises de-identified structured and unstructured data (text and image documents, such as physician notes and pathology reports). The unstructured data were manually curated by trained curators for the ConcertAI Patient360™ Breast Cancer dataset and evaluated for consistency, completeness, and outlier values.

Patients who were female assigned at birth, aged 18–55 years without evidence of being postmenopausal at initial breast cancer diagnosis and exposed to goserelin 3.6 mg or 10.8 mg post-breast cancer diagnosis were included in the study. All patients had at least 360 days of follow-up after the initiation of goserelin or died before 360 days of follow-up. Patients were included in the study regardless of ER, human epidermal growth factor receptor 2 (HER2), and progesterone receptor (PR) status observed in the data. Patients with other primary tumors and those enrolled in a clinical trial during goserelin use were excluded.

Two cohorts were created based on goserelin use: (1) 3.6 mg cohort: patients only receiving goserelin 3.6 mg, and (2) 10.8 mg cohort: patients with any use of goserelin 10.8 mg within 6 months of initial goserelin treatment (includes patients who switched from or to goserelin 3.6 mg). The index date was defined as the date of first goserelin exposure. The study period was defined as the interval between initial breast cancer diagnosis and last activity date, data cutoff, or death (whichever occurred first).

This research was reviewed and determined to be exempt from Institutional Review Board (IRB) oversight by Advarra IRB (Columbia, Maryland). Informed consent was not applicable for this study.

### Study endpoints

The primary outcome was the 12-month rwEFS rate to assess the non-inferiority of 3-month goserelin 10.8 mg versus monthly goserelin 3.6 mg. Secondary endpoints included real-world overall survival (OS) and real-world time to treatment discontinuation (rwTTD). The study endpoints were reported in the overall populations for each cohort and by stage of breast cancer (early-stage/locally advanced or metastatic) at initiation of goserelin.

rwEFS was defined as the time from initiation of goserelin to the first documented date of disease recurrence/disease progression or death from any cause (whichever occurred first). Patients without documented disease recurrence/progression or death were censored on the date of the last activity date. OS was defined as the time from initiation of goserelin to the date of death from any cause. For patients not known to be deceased at the time of analysis, OS was censored at the last date the patient was known to be alive. rwTTD was defined as the time from initiation of goserelin to the last administration without a 60-day gap or death for the 3.6 mg goserelin cohort and without a 180-day gap or death for the 10.8 mg goserelin cohort. This timeframe allows for 2 cycles of each respective dosage to be missed before being considered as discontinued. Patients were censored at last known usage of treatment.

### Statistical analysis

Descriptive statistics were used to summarize patient characteristics and evaluate the differences between the 3.6 mg cohort and the 10.8 mg cohort. Inverse probability of treatment weighting (IPTW) was used to ensure comparability of baseline demographic and clinical characteristics between the two cohorts. IPTW uses the propensity score to balance baseline patient characteristics in the exposed (those who received 10.8 mg goserelin treatment) and unexposed groups (those who received 3.6 mg goserelin treatment) by weighting each individual by the inverse probability of receiving actual treatment. Weights were calculated for each individual as 1/propensity score for the exposed group and 1/(1 − propensity score) for the unexposed group. The standardized difference, which compares the difference in the means between groups, was also reported [15]. Variables included in the IPTW can be found in Online Resource 1. Variables were selected based on the data available in the EMR data.

Non-inferiority testing was conducted for rwEFS rate at 12 months. U.S. FDA guidelines recommend that both statistical and clinical judgment be considered when choosing a non-inferiority margin [16]. Based on the clinical trial results presented by Noguchi et al., the extrapolated 12-month rwEFS was estimated to be 36.2% for the goserelin 3.6 mg cohort and 37.8% for the goserelin 10.8 mg cohort. A power analysis showed a total sample size of 210 patients (105 patients in each cohort) was necessary for the study to demonstrate a non-inferiority margin of 15% with 80% power and an alpha of 0.05. As a result, the non-inferiority margin was set at − 15% between treatment groups based on published oncology clinical trials and the results of the power analysis [17]. The hypothesis for the primary outcome was that goserelin 10.8 mg would be non-inferior to goserelin 3.6 mg at the prespecified margin.

Weighted Kaplan–Meier analyses were conducted for rwEFS, OS, and rwTTD for each cohort. Restricted mean survival time and survival rates at 6 months, 1 year, 3 years, and 5 years were calculated for rwEFS and OS [18]. The proportion of patients who remained on the treatment at 6 months, 12 months, 18 months, and 24 months was also reported.

## Results

### Patient characteristics

A total of 698 patients were included in the study, with 575 patients in the goserelin 3.6 mg cohort and 123 patients in the goserelin 10.8 mg cohort. In the goserelin 10.8 mg cohort, 35 (28.5%) patients switched to or from goserelin 3.6 mg and received goserelin 10.8 mg within 6 months of initial goserelin treatment.

After weighting, the 3.6 mg cohort had a median age of 41 years, and 70.3% of patients were White (Table 1). A majority of patients (68.3%) had infiltrating ductal carcinoma histology, and 61.0% of patients had early-stage/locally advanced breast cancer at initial goserelin treatment. The 10.8 mg cohort had a median age of 42 years, and 69.9% of patients were White. Most patients (70.2%) in the 10.8 mg cohort had infiltrating ductal carcinoma histology, and 61.0% of patients had early-stage/locally advanced breast cancer at initial goserelin treatment. Online Resource 2 presents the Love plot of the standardized differences for the unweighted and weighted variables. The median follow-up time from the index date to the end of the record for the 3.6 mg cohort was 37.1 months and 36.3 months for the 10.8 mg cohort.

**Table 1:** Patient Characteristics Pre- and Post-Weighting by Goserelin Dosage

	Pre-Weighting		Post-Weighting		
Variable	Goserelin 3.6 mg *n* = 575	Goserelin 10.8 mg^a^ *n* = 123	Goserelin 3.6 mg *n* = 700	Goserelin 10.8 mg^a^ *n* = 670	Standardized Difference
Age at initial breast cancer diagnosis, median (min, max) (years)	41 (24–55)	41 (22–55)	41 (24–55)	42 (22–55)	0.0521
Race, *n* (%)					0.02884
White	418 (72.7)	74 (60.2)	492 (70.3)	468 (69.9)	
Black or African American	77 (13.4)	26 (21.1)	101 (14.5)	103 (15.3)	
Other or unknown race	80 (13.9)	23 (18.7)	106 (15.2)	99 (14.8)	
Stage at initial breast cancer diagnosis, *n* (%)					0.4676
Stage 0 + Stage I	127 (22.1)	26 (21.1)	153 (21.8)	158 (23.5)	
Stage II	196 (34.1)	48 (39.0)	246 (35.2)	229 (34.2)	
Stage III	110 (19.1)	19 (15.4)	129 (18.5)	114 (17.0)	
Stage IV	86 (15.0)	21 (17.1)	106 (15.2)	90 (13.4)	
Unknown	56 (9.7)	9 (7.3)	66 (9.4)	79 (11.8)	
Comorbidities at initial breast cancer diagnosis, *n* (%)					
Chronic obstructive pulmonary disease, unspecified	29 (5.0)	3 (2.4)	32 (4.6)	37 (5.5)	
Type 2 diabetes mellitus without complications	24 (4.2)	1 (< 1)	31 (4.4)	7 (1.0)	
Autoimmune disease	10 (1.7)	2 (1.6)	11 (1.6)	16 (2.4)	
Surgical procedures prior to initial goserelin therapy, *n* (%)					0.04397
Mastectomy	309 (53.7)	69 (56.1)	381 (54.4)	351 (52.3)	
Excision	81 (14.1)	15 (12.2)	95 (13.6)	98 (14.6)	
Both mastectomy and excision	66 (11.5)	18 (14.6)	84 (12.0)	80 (12.0)	
No surgery	119 (20.7)	21 (17.1)	140 (19.9)	141 (21.1)	
Timing of initial goserelin therapy, *n* (%)					0.00109
Early-stage/locally advanced breast cancer, prior to metastatic diagnosis (if any)	349 (60.7)	77 (62.6)	427 (61.0)	409 (61.0)	
At or after metastatic diagnosis	226 (39.3)	46 (37.4)	273 (39.0)	261 (39.0)	
Histology at initial breast cancer diagnosis, *n* (%)					0.18494
Infiltrating duct	393 (68.3)	81 (65.9)	478 (68.3)	470 (70.2)	
Infiltrating lobular	46 (8.0)	4 (3.3)	50 (7.1)	27 (4.1)	
Infiltrating duct and lobular	28 (4.9)	8 (6.5)	36 (5.2)	45 (6.8)	
Carcinoma in situ + Other/Unknown	108 (18.8)	30 (24.4)	136 (19.4)	127 (18.9)	
HER2 status, *n* (%)					0.10448
Positive	87 (15.1)	20 (16.3)	106 (15.1)	81 (12.0)	
Low	211 (36.7)	44 (35.8)	257 (36.7)	229 (34.3)	
Negative	253 (44.0)	51 (41.5)	302 (43.2)	324 (48.3)	
Equivocal/Conflict/Unable to confirm/Unknown	24 (4.2)	8 (6.5)	35 (5.0)	36 (5.4)	
PR status, *n* (%)					0.12908
Positive	486 (84.5)	102 (82.9)	586 (83.8)	534 (79.7)	
Negative	76 (13.2)	16 (13.0)	92 (13.2)	111 (16.5)	
Conflict/Unknown	13 (2.3)	5 (4.1)	21 (3.0)	25 (3.8)	

^a^Patients who received goserelin 10.8 mg and switched to or from 3.6 mg were included in the 10.8 mg category

Data source: ConcertAI Patient360™ Breast Cancer dataset

Study population: Patients who were female assigned at birth, aged 18–55 years without evidence of being postmenopausal at initial breast cancer diagnosis and exposed to goserelin 3.6 mg or 10.8 mg post-breast cancer diagnosis were included in the study. All patients had at least 360 days of follow-up after the initiation of goserelin or died before 360 days of follow-up. Two cohorts were created based on goserelin use: 1) 3.6 mg cohort: patients only receiving goserelin 3.6 mg, and 2) 10.8 mg cohort: patients with any use of goserelin 10.8 mg within 6 months of initial goserelin treatment (includes patients who switched from or to goserelin 3.6 mg)

Index date: The date of first goserelin exposure

The covariates used in weighting are presented in Online Resource 1

HER2: human epidermal growth factor receptor 2; Max: maximum, Min: minimum; PR: progesterone receptor

### Non-inferiority test

**Fig. 1:**
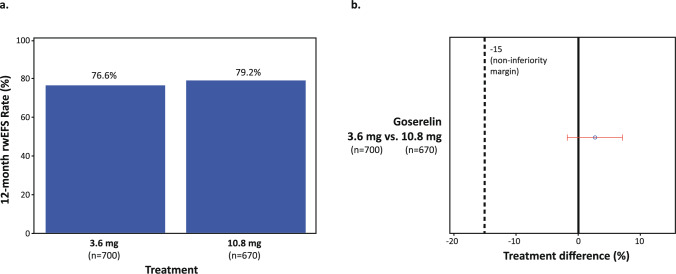
Weighted Bar Graph (**a**) and Forest Plot (**b**) of 12-Month rwEFS Rate by Dosage of Goserelin. Results after inverse probability of treatment weighting. The covariates used in weighting are presented in Online Resource 1. rwEFS: real-world event-free survival

The 12-month rwEFS rates were 76.6% for the 3.6 mg cohort and 79.2% for the 10.8 mg cohort, with a treatment difference of 2.7% (95% CI − 1.8%, 7.0%) (Table 2, Fig. 1). This result supports the hypothesis that goserelin 10.8 mg is non-inferior to goserelin 3.6 mg at the prespecified − 15% margin.

**Table 2:** Weighted Non-Inferiority^a^ Analysis of 12-Month rwEFS Rate by Dosage of Goserelin

Non-Inferiority Test	Goserelin 3.6 mg	Goserelin 10.8 mg^b^	Treatment Difference
Overall			
n	**700**	**670**	
12-month rwEFS rate	76.6%	79.2%	2.7%
95% CI	72.8%, 79.9%	68.2%, 86.8%	−1.8%, 7.0%
Early-stage/locally advanced breast cancer at index date			
n	**427**	**409**	
12-month rwEFS rate	93.2%	90.9%	−2.3%
95% CI	89.9%, 95.4%	77.7%, 96.4%	−6.0%, 1.4%
Metastatic breast cancer at index date			
n	**273**	**261**	
12-month rwEFS rate	50.6%	61.0%	10.4%
95% CI	43.8%, 57.0%	41.2%, 75.9%	2.0%, 18.9%

Time origin: Start date of initial goserelin treatment (index date)

Terminal event: Date of disease progression, recurrence, or death

Censoring: End of record

Results after inverse probability of treatment weighting. The covariates used in weighting are presented in Online Resource 1

^a^Non-inferiority for rwEFS was based on a prespecified margin of 15% between treatment groups using a 2-sided 95% CI

^b^Patients who received goserelin 10.8 mg and switched to or from 3.6 mg were included in the 10.8 mg category

CI: confidence interval, rwEFS: real-world event-free survival

For patients with early-stage/locally advanced breast cancer at the time of initial goserelin treatment, the 12-month rwEFS rate was 93.2% for the 3.6 mg cohort and 90.9% for the 10.8 mg cohort, with a treatment difference of − 2.3% (95% CI − 6.0%, 1.4%). For patients with metastatic breast cancer at the time of initial goserelin treatment, the 12-month rwEFS rate was 50.6% for the 3.6 mg cohort and 61.0% for the 10.8 mg cohort, with a treatment difference of 10.4% (95% CI 2.0%, 18.9%). These results suggested that goserelin 10.8 mg was non-inferior to goserelin 3.6 mg at the prespecified − 15% margin regardless of stage of disease at time of initiation.

### Real-world event-free survival

**Fig. 2:**
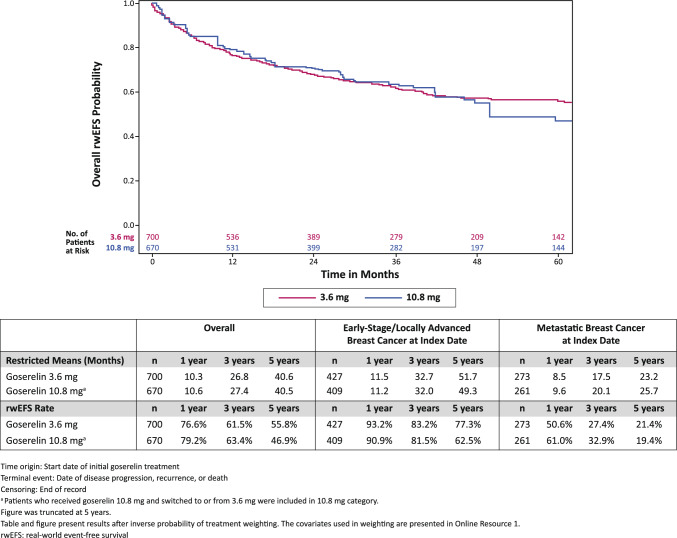
Weighted Kaplan–Meier Analysis of rwEFS by Dosage of Goserelin

Kaplan–Meier analysis showed patients in the 3.6 mg cohort had a rwEFS rate at 1 year of 76.6% and 79.2% for the 10.8 mg cohort (Fig. 2). The rwEFS rate at 3 years for the 3.6 mg cohort was 61.5% and 63.4% for the 10.8 mg cohort. The rwEFS rate at 5 years for the 3.6 mg cohort was 55.8% and 46.9% for the 10.8 mg cohort. The restricted means for each cohort are presented in Fig. 2.

For patients with early-stage/locally advanced breast cancer at the time of initial goserelin treatment, the rwEFS rate at 1 year for the 3.6 mg cohort was 93.2% and 90.9% for the 10.8 mg cohort. The rwEFS rate at 3 years for the 3.6 mg cohort was 83.2% and 81.5% for the 10.8 mg cohort. The rwEFS rate at 5 years for the 3.6 mg cohort was 77.3% and 62.5% for the 10.8 mg cohort. The restricted means for patients with early-stage/locally advanced breast cancer are presented in Fig. 2.

For patients with metastatic breast cancer at the time of initial goserelin treatment, the rwEFS rate at 1 year for the 3.6 mg cohort was 50.6% and 61.0% for the 10.8 mg cohort. The rwEFS rate at 3 years for the 3.6 mg cohort was 27.4% and 32.9% for the 10.8 mg cohort. The rwEFS rate at 5 years for the 3.6 mg cohort was 21.4% and 19.4% for the 10.8 mg cohort. The restricted means for patients with metastatic breast cancer are presented in Fig. 2.

### Real-world overall survival

**Fig. 3:**
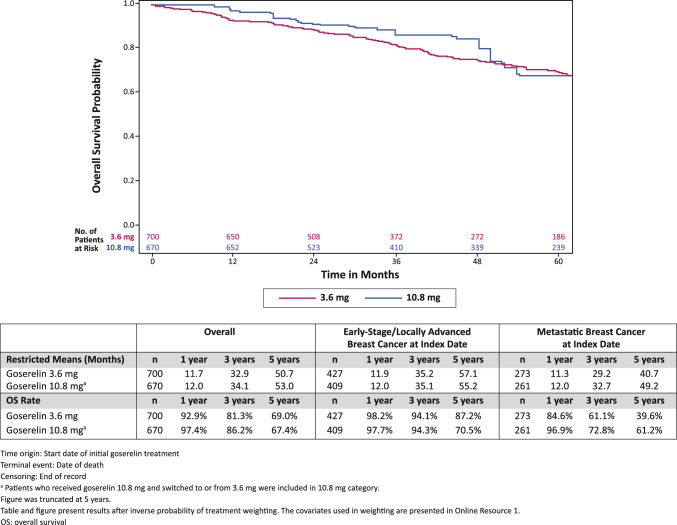
Weighted Kaplan–Meier Analysis of OS by Dosage of Goserelin

For OS, the rate at 1 year for the 3.6 mg cohort was 92.9% and 97.4% for the 10.8 mg cohort (Fig. 3). The OS rate at 3 years for the 3.6 mg cohort was 81.3% and 86.2% for the 10.8 mg cohort. The OS rate at 5 years for the 3.6 mg cohort was 69.0% and 67.4% for the 10.8 mg cohort. The restricted means for each cohort are presented in Fig. 3.

For patients with early-stage/locally advanced breast cancer at the time of initial goserelin treatment, the OS rate at 1 year for the 3.6 mg cohort was 98.2% and 97.7% for the 10.8 mg cohort. The OS rate at 3 years for the 3.6 mg cohort was 94.1% and 94.3% for the 10.8 mg cohort. The OS rate at 5 years for the 3.6 mg cohort was 87.2% and 70.5% for the 10.8 mg cohort. The restricted means for patients with early-stage/locally advanced breast cancer are presented in Fig. 3.

For patients with metastatic breast cancer at the time of initial goserelin treatment, the OS rate at 1 year for the 3.6 mg cohort was 84.6% and 96.9% for the 10.8 mg cohort. The OS rate at 3 years for the 3.6 mg cohort was 61.1% and 72.8% for the 10.8 mg cohort. The OS rate at 5 years for the 3.6 mg cohort was 39.6% and 61.2% for the 10.8 mg cohort. The restricted means for patients with metastatic breast cancer are presented in Fig. 3.

### Real-world time to discontinuation

**Fig. 4:**
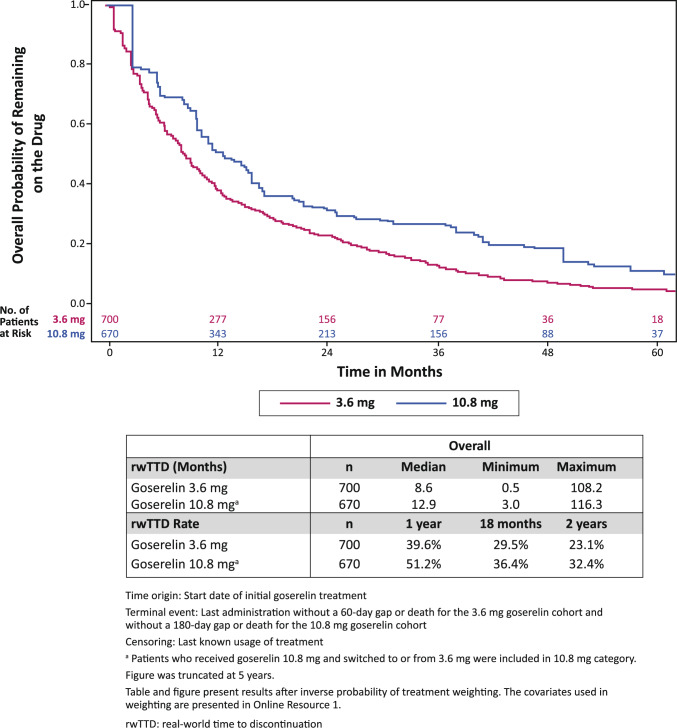
Weighted Kaplan–Meier Analysis of rwTTD by Dosage of Goserelin

The proportion of patients who remained on the initial goserelin treatment at 1 year for the 3.6 mg cohort was 39.6% and 51.2% for the 10.8 mg cohort (Fig. 4). The proportion of patients who remained on the initial goserelin at 2 years for the 3.6 mg cohort was 23.1% and 32.4% for the 10.8 mg cohort. The median rwTTD for the 3.6 mg cohort was 8.6 months and 12.9 months for the 10.8 mg cohort. For patients with early-stage/locally advanced breast cancer at the time of initial goserelin treatment, the median rwTTD for the 3.6 mg cohort was 8.8 months and 15.7 months for the 10.8 mg cohort. For patients with metastatic breast cancer at the time of initial goserelin treatment, the median rwTTD for the 3.6 mg cohort was 8.4 months and 10.0 months for the 10.8 mg cohort.

## Discussion

This real-world analysis indicated that goserelin 10.8 mg was non-inferior to goserelin 3.6 mg among premenopausal patients with breast cancer in terms of 12-month rwEFS rate, regardless of the stage of breast cancer at initiation of goserelin treatment. The OS rates were similar among both groups at 1, 3, and 5 years. At 1 year, almost 40% of patients in the goserelin 3.6 mg cohort remained on goserelin treatment while more than half of patients remained on goserelin in the goserelin 10.8 mg cohort.

This study adds to the literature by examining the clinical outcomes of goserelin 10.8 mg versus goserelin 3.6 mg in the real-world setting, which to our knowledge has not been previously examined. The study also used IPTW to balance patient cohorts while observing the differences between dosages, which was necessary because goserelin 3.6 mg has been approved in the treatment of breast cancer in the U.S. for decades and goserelin 10.8 mg is not currently approved in the U.S. for breast cancer.

The results of the non-inferiority analysis are similar to those reported by Noguchi and colleagues, who reported a treatment difference between goserelin 3.6 mg and goserelin 10.8 mg of 1.3% for the 24-week progression-free survival rate in a Phase 3 clinical trial for patients with advanced-stage breast cancer [14]. Our study, which reported 12-month rwEFS rates, showed a treatment difference of 2.7% between the two groups overall and 10.4% for patients with metastatic breast cancer at initial goserelin treatment. The observed non-inferiority of goserelin 10.8 mg in this real-world study may support the use of goserelin 10.8 mg as an alternative treatment option in this patient population.

We observed a longer rwTTD for patients in the goserelin 10.8 mg cohort compared with patients in the goserelin 3.6 mg cohort. A possible reason is the convenience of 3-month intervals (goserelin 10.8 mg) versus 1-month intervals (goserelin 3.6 mg) for treatment administration, making it more practical for patients to continue treatment longer. There may be other factors that could influence a patient’s choice of dosing schedule such as cost, insurance coverage, gauge of needle (larger size of 10.8 mg implant which may lead to injection discomfort), or tolerance to the amount of medication. The longer time to rwTTD for patients who received goserelin 10.8 mg needs to be confirmed in future studies as this outcome was not comparative in nature.

The observed 3-year OS results were slightly lower compared with those reported by Wu and colleagues [9]. For patients with metastatic breast cancer at the time of initial goserelin treatment, our study found that the goserelin 3.6 mg cohort had a 3-year OS rate of 61.1% and those in the goserelin 10.8 mg cohort had a rate of 72.8%. Wu and colleagues reported a 3-year OS rate of 76.6% for patients with advanced-stage breast cancer who received goserelin 3.6 mg in combination with endocrine therapy in China. However, our study included a much larger sample size (698 patients versus 40 patients), and it also included patients who developed distant metastases after initial treatment or who received goserelin 3.6 mg before disease progression. Additionally, the management of patients with HER2-positive disease may differ in the U.S.

The strengths of this study include the use of IPTW to help balance the two cohorts for comparison. The study also included a large sample of patients with breast cancer from a geographically diverse area of the U.S. and had the ability to include patients treated with goserelin 10.8 mg, even though this dosage is not FDA approved in the U.S. IPTW was used to mitigate any potential differences in patient characteristics in those who received goserelin 10.8 mg off-label compared with patients who received goserelin 3.6 mg on-label. Additionally, the study only included patients with at least 360 days of follow-up or death within 360 days. This was necessary to accurately estimate the main study objective, which was the 12-month rwEFS rate. Finally, this study used data created through manual human review, including disease recurrence, which was the main endpoint of this study.

There are several limitations to this study. First, the retrospective nature of this study lends itself to inherent challenges such as data missingness and potential misclassification, and findings should be interpreted with caution. However, potential biases were reduced using both structured and human-reviewed unstructured EMR data. Next, while IPTW was used to help balance the cohorts, other unmeasurable differences may exist between the two cohorts. The study also included patients in the goserelin 10.8 mg cohort who switched to and from goserelin 3.6 mg. Only 28.5% of the goserelin 10.8 mg cohort used goserelin 3.6 mg, and the inclusion criteria required the patients to have started goserelin 10.8 mg within 6 months of initial goserelin treatment. Additionally, our study could not differentiate whether goserelin was prescribed for ovarian function preservation during curative chemotherapy or ovarian function suppression. Finally, this study reflected treatment practice patterns primarily within community oncology practices in the U.S. that are part of the ConcertAI network. As a result, the results may not be generalizable to patients treated in academic settings or outside the U.S.

## Conclusion

This U.S.-based real-world analysis indicated that 3-month goserelin 10.8 mg is non-inferior to monthly 3.6 mg in premenopausal patients with breast cancer in terms of 12-month rwEFS rate. These findings are similar to those previously reported in clinical trials and may support the use of the 3-month goserelin 10.8 mg implant as an alternative treatment option for this patient population.

## Supplementary Information

Below is the link to the electronic supplementary material.

Supplementary file1 (DOCX 112 KB)

## Data Availability

The data that support the findings of this study are available from ConcertAI, LLC but restrictions apply to the availability of these data, which were used under license for the current study, and so are not publicly available. Data are, however, available from ConcertAI, LLC upon reasonable request (https://www.concertai.com/contact-us/).

## Declarations

### Previous publication

Part of this research was presented at the San Antonio Breast Cancer Symposium (SABCS) (December 10-13, 2024).
