# Supplementary material for: Goserelin 3-month depot shows non-inferiority to the monthly formulation in U.S. patients with premenopausal breast cancer: a real-world evidence study
Source: Breast Cancer Res Treat. 2025 Mar 6;211(2):409–19. doi: 10.1007/s10549-025-07656-z (PMC12006218; doi:10.1007/s10549-025-07656-z)
Supplement: Supplementary file 1 — Supplementary file1 (DOCX 112 KB) [file 10549_2025_7656_MOESM1_ESM.docx]

**Supplementary Information**

Article title: Goserelin 3-month depot shows noninferiority to the monthly formulation in US patients with premenopausal breast cancer: A real-world evidence study

Journal: *Breast Cancer Research and Treatment*

Authors: Kelly E. McCann^*^, Noran Osman, Joan Cannon, Lonnie Brent, Yuexi Wang, Jon Tepsick, Prithviraj Vikramsinh Mandora, Vincent Miller, Nancy Martin, Virginia G. Kaklamani

*Corresponding author:

Kelly E. McCann, MD, PhD

Assistant Clinical Professor

David Geffen School of Medicine

University of California Los Angeles

Los Angeles, CA, USA

Email: [kmccann@mednet.ucla.edu](mailto:kmccann@mednet.ucla.edu)

| **Online Resource 1. List of Covariates for Inverse Probability of Treatment Weighting** |
| --- |
| - Age at initial breast cancer diagnosis |
| - - Continuous variable |
| - Race |
| - - White, Black, and other/unknown |
| - Ethnicity |
| - - Hispanic/Latino, not Hispanic/Latino, unknown |
| - U.S. geographic region |
| - - Midwest, Northeast, South, West, unknown |
| - Stage at initial breast cancer diagnosis |
| - - Stage 0 +1, stage 2, stage 3, stage 4, unknown |
| - Histology |
| - - Infiltrating duct, infiltrating lobular, infiltrating duct and lobular, carcinoma in situ/other/unknown |
| - Tumor grade |
| - - Grade 1, Grade, 2, Grade 3, Grade X + unknown |
| - Eastern Cooperative Oncology Group performance status at initial breast cancer diagnosis |
| - - 0, 1, 2+, unknown |
| - Charlson Comorbidity Index at initial breast cancer diagnosis |
| - - 0, 1+ |
| - Body mass index at initial diagnosis |
| - - <18.5, 18.5–24.9, 25–30.0, >30 |
| - Surgery prior to initial goserelin |
| - - Mastectomy, excision, both, no surgery |
| - Timing of initial goserelin |
| - - Early-stage/locally advanced breast cancer, prior to metastatic diagnosis (if any) |
| - - On or after metastatic disease |
| - Use of aromatase inhibitor prior to initial goserelin |
| - Progesterone receptor status |
| - - Positive, negative, unknown + conflict |
| - HER2 status |
| - - Positive, low, negative, unknown + unable to confirm + equivocal + conflict |
| - BRCA status |
| - - BRCA wild type (negative for BRCA1 and BRCA2); positive for either BRCA1/2; other/unknown (including those who were negative for only 1 of the 3) |
| - MKI67 (percent of positive cells) |
| - - <5%, 5–20%, >20%, unknown |
|  |

**Online Resource 2. Love Plot Displaying Balance for Unweighted and Weighted Covariates**


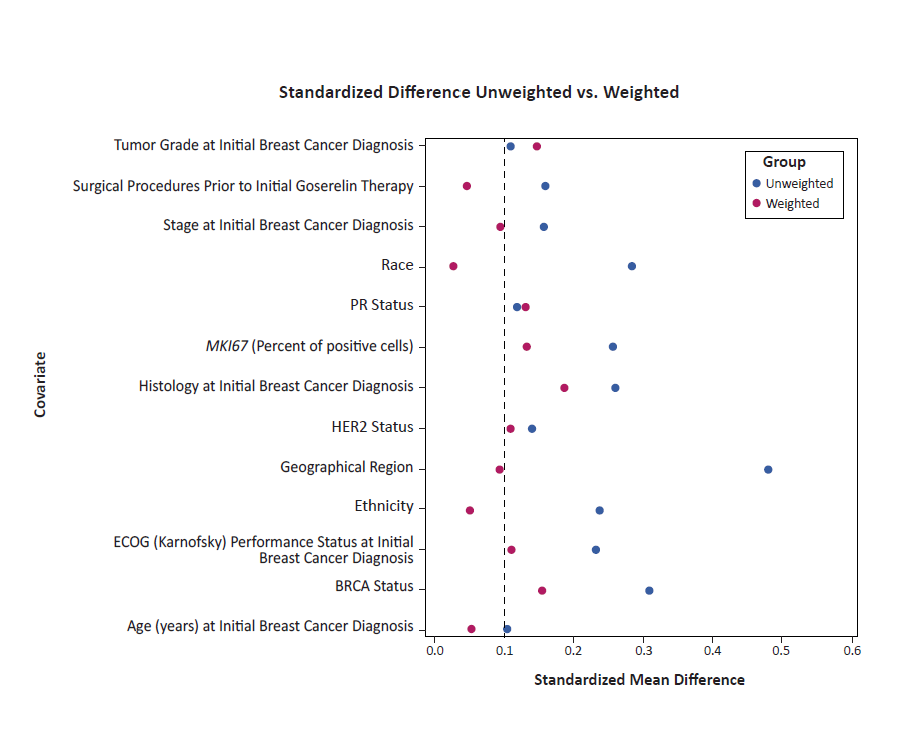


ECOG: Eastern Cooperative Oncology Group, PR: progesterone receptor
